# Neuron-specific enolase serum levels in COVID-19 are related to the severity of lung injury

**DOI:** 10.1371/journal.pone.0251819

**Published:** 2021-05-19

**Authors:** Erika Cione, Antonio Siniscalchi, Pietro Gangemi, Lucio Cosco, Manuela Colosimo, Federico Longhini, Filippo Luciani, Giovambattista De Sarro, G&SP Working Group, Liberato Berrino, Bruno D’Agostino, Luca Gallelli

**Affiliations:** 1 Department of Pharmacy, Health and Nutritional Sciences-Department of Excellence 2018-2020, University of Calabria, Rende, Cosenza, Italy; 2 Department of Neurology, Annunziata Hospital, Cosenza, Italy; 3 Operative Unit of Clinical Chemistry Laboratory, Pugliese Ciaccio Hospital, Catanzaro, Italy; 4 Department of Infectious Disease, Pugliese Ciaccio Hospital, Catanzaro, Italy; 5 Department of Microbiology and Virology, Pugliese Ciaccio Hospital, Catanzaro, Italy; 6 Department of Medical and Surgical Science, Operative Unit of Anesthesiology and Reanimation, School of Medicine, University of Catanzaro, Catanzaro, Italy; 7 Department of Infectious Disease, Annunziata Hospital, Cosenza, Italy; 8 Department of Health Science, School of Medicine, Operative Unit of Clinical Pharmacology, Mater Domini University Hospital, University of Catanzaro, Catanzaro, Italy; 9 Department of Health Science, School of Medicine, Research Center FA@UNICZ, University of Catanzaro, Catanzaro, Italy; 10 Department of Experimental Medicine L. Donatelli, Section of Pharmacology, School of Medicine, University of Campania Luigi Vanvitelli, Naples, Italy; Defense Threat Reduction Agency, UNITED STATES

## Abstract

The multifunctional role of neuron-specific enolase (NSE) in lung diseases is well established. As the lungs are greatly affected in COVID-19, we evaluated serum NSE levels in COVID-19 patients with and without dyspnea. In this study, we evaluated both SARS-CoV-2-infected and uninfected patients aged >18 years who were referred to hospitals in Catanzaro, Italy from March 30 to July 30, 2020. Epidemiological, clinical, and radiological characteristics, treatment, and outcome data were recorded and reviewed by a trained team of physicians. In total, 323 patients (178 men, 55.1% and 145 women, 44.9%) were enrolled; of these, 128 were COVID-19 patients (39.6%) and 195 were control patients (60.4%). Westergren’s method was used to determine erythroid sedimentation rate. A chemiluminescence assay was used for measurement of interleukin-6, procalcitonin, C-reactive protein, and NSE. We detected significantly higher NSE values (P<0.05) in COVID-19 patients than in controls. Interestingly, within the COVID-19 group, we also observed a further significant increase in dyspnea (Dyspnea Scale and Exercise score: 8.2 ± 0.8; scores ranging from 0 to 10, with higher numbers indicating very severe shortness of breath). These data provide the background for further investigations into the potential role of NSE as a clinical marker of COVID-19 progression.

## 1. Introduction

The high mortality rate caused by rapid spread of the severe acute respiratory syndrome coronavirus 2 (SARS-CoV-2) is constantly increasing. Therefore, research on soluble factors related to the diagnosis, severity, and management of patients infected with SARS-CoV-2 is urgently needed [1]. Neuron-specific enolase (NSE) is a glycolytic enzyme that is localized in the cytoplasm of neurons and neuroendocrine cells. It is found primarily in the amine precursor uptake and decarboxylation (APUD) lineage, such as in the pituitary, thyroid, pancreas, intestine, and lung [2]. NSE is used as a tumor marker in the diagnosis, prognosis, and follow-up of small cell lung cancer (SCLC), and its serum levels differ significantly according to tumor size, disease stage, and metastasis [3]. The involvement of NSE in lung diseases other than cancer has also been documented. Clinical evidence highlights the role of NSE in the diagnosis, treatment, and monitoring of both acute and chronic lung injuries [4, 5], solitary pulmonary nodules [6], and infectious lung diseases such as tuberculosis [7]. In pulmonary tuberculosis with no neoplastic pathology, the sensitivity of serum NSE was high in smear-negative tuberculosis with small amounts of bacilli [7]. Even in children diagnosed with acute miliary tuberculosis, NSE levels in the cerebrospinal fluid (CSF) and serum were significantly elevated [8]. NSE levels in both serum and CSF displayed the same fluctuation behavior. To ensure optimal environments for their replication and spread, viruses can alter many host cell metabolic pathways, including aerobic glycolysis [9]. Since the lungs are significantly affected by coronavirus disease 2019 (COVID-19), we evaluated, for the first time, the serum NSE levels in SARS-CoV-2 infected patients with and without dyspnea.

## 2. Methods

### 2.1 Population

We performed a pilot observational study on SARS-CoV-2 infected patients with and without dyspnea who were referred to the emergency department or intensive care unit of the Pugliese-Ciaccio Hospital in Catanzaro, Italy from March 30 to July 30, 2020. We also enrolled patients hospitalized for all causes but without SARS-COV-2 infection (control group). This study was a part of the clinical trials recorded at clinicaltrials.gov (NCT04322513) and was approved by the local Ethics Committee (Calabria Centro). This study was conducted in compliance with the Institutional Review Board/Human Subjects Research Committee requirements, the Declaration of Helsinki, and the Guidelines for Good Clinical Practice criteria. Before the beginning of the study, enrolled patients or their legal guardians provided informed consent.

The inclusion criteria were as follows: Patients of both sexes, >18 years, with symptoms of acute airway disease (e.g., cough, sore throat, breathing difficulties), fever, muscle pain, sudden anosmia or ageusia, and a RT-PCR nasopharyngeal swab positive for SARS-CoV-2 infection.

The exclusion criteria were as follows: Patients who did not provide informed consent, patients with a clinical history of previous systemic diseases (e.g., cancer, epilepsy, respiratory or cardiovascular failure, or obesity), and patients without respiratory symptoms.

The endpoints were as follows: The first endpoint was a statistically significant difference (P<0.05) in serum NSE levels in patients infected with SARS-CoV-2 when compared to the control group. The second endpoint was a statistically significant difference (P<0.05) in serum NSE levels in SARS-CoV-2-infected patients with dyspnea compared to SARS-CoV-2-infected patients without dyspnea.

### 2.2 Data collection and clinical biochemistry assays

The medical records of patients from March 30, 2020 to July 30, 2020 were analyzed. Epidemiological, clinical, and radiological characteristics, as well as treatment and outcome data were obtained at the time of enrollment and reviewed by a trained team of physicians. The information recorded included demographic data, medical history, exposure history, underlying comorbidities, symptoms, signs, laboratory findings, chest X-ray (CXR) findings, related computer tomography (CT) findings, and pharmacological treatment. The date of disease onset was defined as the day when symptoms appeared. Westergren’s method was used to evaluate the erythrocyte sedimentation rate (ESR). Clinical biochemistry data regarding soluble serum levels factors were detected by chemiluminescence assay (CLIA) for interleukin-6 (IL-6), procalcitonin, C-reactive protein (CRP), and NSE [10].

### 2.3 Real-Time Polymerase Chain Reaction (RT-PCR) assay for SARS-CoV-2

Nasopharyngeal swab samples were collected to extract SARS-CoV-2 RNA from patients suspected of having COVID-19. After collection, the swabs were placed into a collection tube with 150 μL of virus preservation solution, and total RNA was extracted within 2 h using automated nucleic acid (NA) extraction and PCR (Nimbus, Seegene Inc., Korea). The suspension was used for the RT-PCR assay of the SARS-CoV-2 RNA. Two target genes for SARS-CoV-2, RNA-dependent RNA polymerase (RdRP) and nucleocapsid protein (N), were simultaneously amplified and tested during the RT-PCR assay using a SARS-CoV-2 nucleic acid detection kit according to the manufacturer’s protocol (Allplex^™^ 2019-nCoV Assay, Seegene Inc., Korea). RT-PCR assay was performed under the following conditions: incubation at 50 °C for 15 min and 95 °C for 5 min, 45 cycles of denaturation at 94 °C for 15 s, and extension and collection of fluorescence signal at 55 °C for 45 s. A cycle threshold value (Ct value) of less than 39 was defined as positive. According to the World Health Organization Berlin’s recommendation, these diagnostic criteria were based on the recommendations of the Italian Health Institute.

### 2.4 Statistical analysis

All data were analyzed using the SPSS statistical program (SPSS Inc., Chicago, USA) by evaluating arithmetic characteristics such as mean, geometric mean, and standard deviation (SD). Data parameters were checked for normality using the Shapiro-Wilk normality test. Data were analyzed using one-way analysis of variance and the χ^2^ test. Bonferroni correction was used to estimate the P-value for the multiple logistic model to counteract multiple testing effects. The threshold for statistical significance was set at P<0.05. We defined and labeled this study as exploratory; therefore, we did not perform a power calculation.

## 3. Results

### 3.1 Clinical and NSE data of SARS-CoV-2 infected patients

**Fig 1:**
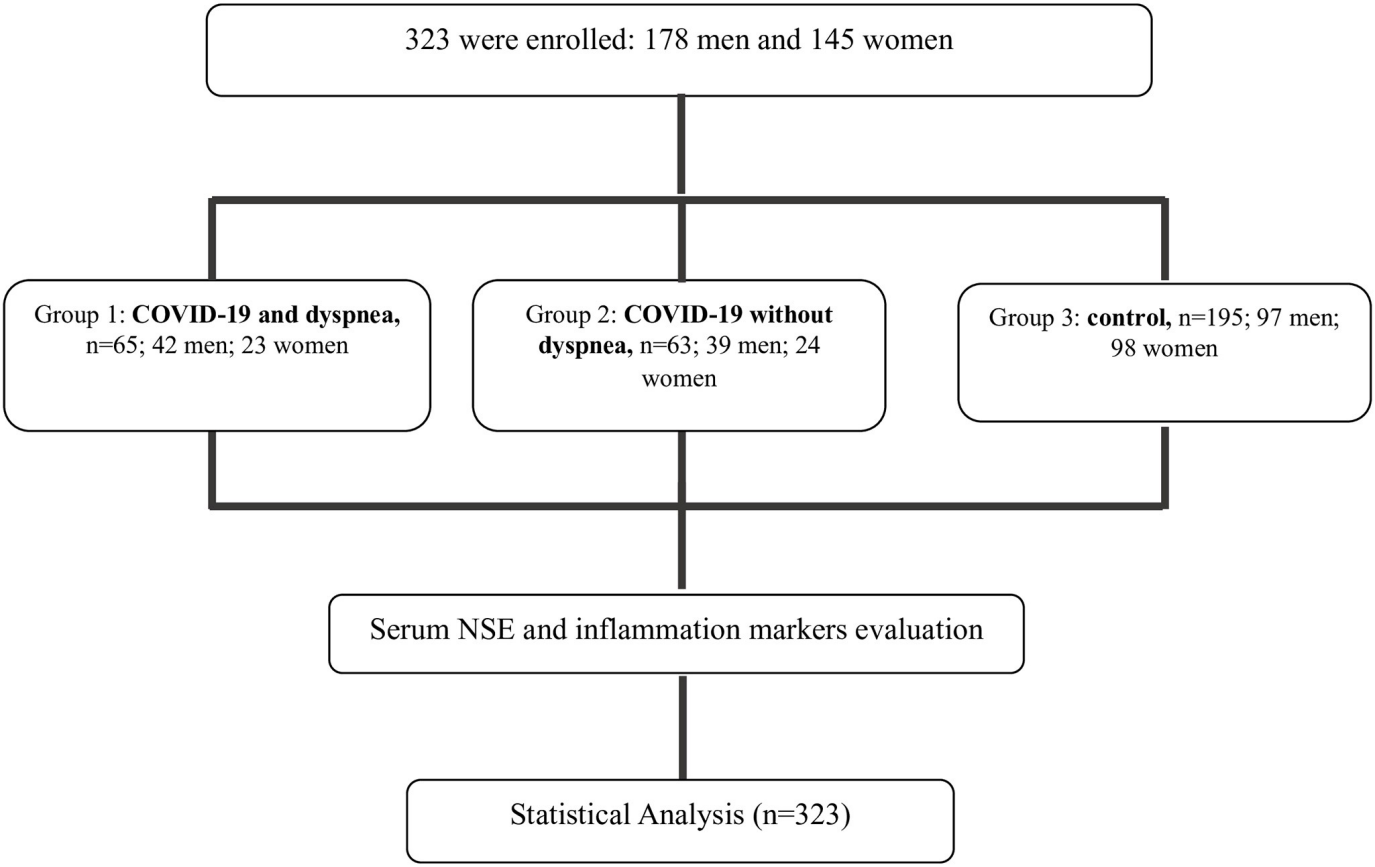
Consort enrollment of patients enclosed in the study.

During the study, we enrolled 323 patients (178 men, 55.1%; 45 women, 44.9%); 128 were enrolled in the COVID-19 group (39.6%) (Groups 1 and 2) and 195 were enrolled in the control group (60.4%) (Group 3). Participants were aged 20 to 68 years and did not have a clinical history of previous systemic diseases (e.g., cancer, epilepsy, respiratory or cardiovascular failure, obesity) (Fig 1).

**Fig 2:**
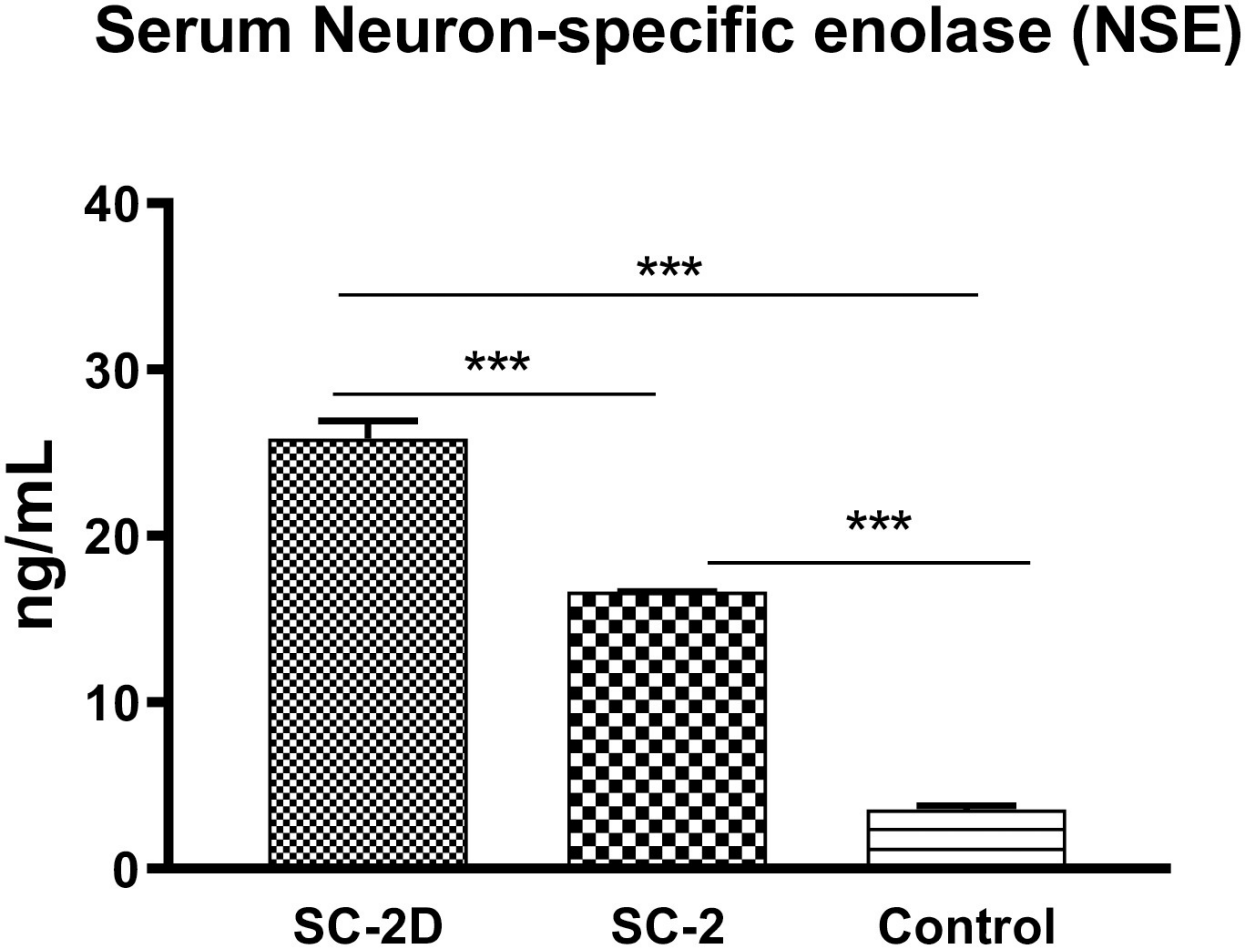
Neuron-Specific Enolase (NSE) serum levels. Clinical biochemistry data on NSE in Group 1: SC-2D, Group 2: SC-2, and Group 3: Control. Results are shown as mean ± SD. The analysis of variance test was performed to evaluate the differences between groups. ***P<0.01. P values remained significant after Bonferroni comparisons (P<0.01).

The enrolled patients were further divided into two groups: **Group 1**, consisting of patients infected with SARS-CoV-2 with dyspnea (SC-2D), and **Group 2**, consisting of patients infected with SARS-CoV-2 without dyspnea (SC-2). A third group (**Group 3**) was included as a control. NSE levels were determined in serum by CLIA. **Group 1** consisted of 65 patients (50.8%) with a mean age of 49.14 ± 11.29. In this group, clinical evaluation revealed the presence of anosmia, fever, dry cough, and dyspnea. The mean Dyspnea Scale and Exercise (DSE) score was 8.2 ± 0.8 (DSE scores range from 0 to 10; high values indicate very severe shortness of breath) (Table 1). Neurological examination excluded the presence of neurological diseases, while clinical biochemistry data reported elevated NSE levels of 25.85 ± 8.64. **Group 2** included 63 patients (49.2%), with a mean age of 50.10 ± 10.50 years, who had similar characteristics to those in Group 1 but without dyspnea. In these patients, NSE levels (16.64 ± 0.15 ng/mL) were significantly lower than those in Group 1 (P<0.01) but higher than those in Group 3 (P<0.01) (Table 1 and Fig 2). Finally, **Group 3** consisted of 195 patients (mean age 49.91 ± 11.30 years) without SARS-CoV-2 infection and with similar characteristics to Group 2. In this group, NSE levels (3.56 ± 2.9 ng/mL) were significantly lower than those in Groups 1 and 2 (P<0.01) (Fig 2).

**Table 1:** Clinical characteristics of enrolled patients.

Variable	Group 1: SC-2D (n = 65)	Group 2: SC-2 (n = 63)	Group 3: Control (n = 195)
Age (mean ± SD)	49.14 ± 14.89	50.1 ± 13.5	49.91 ± 14.6
Male	42	39	97
Female	23	24	98
Medical history and risk factors for respiratory diseases	None	None	None
Intensive care unit admission	9	10	--
Discharged home	--	--	--
Dyspnea Scale and Exercise	8.2 ± 0.8	Not present	Not present
Anosmia	58	59	0
Fever	65	63	0
Dry Cough	62	59	0

Group 1 SC-2D: SARS-CoV-2 infected patients with dyspnea; Group 2 SC-2: SARS-CoV-2 infected patients without dyspnoea; Group 3: Non-infected control patients.

**Fig 3:**
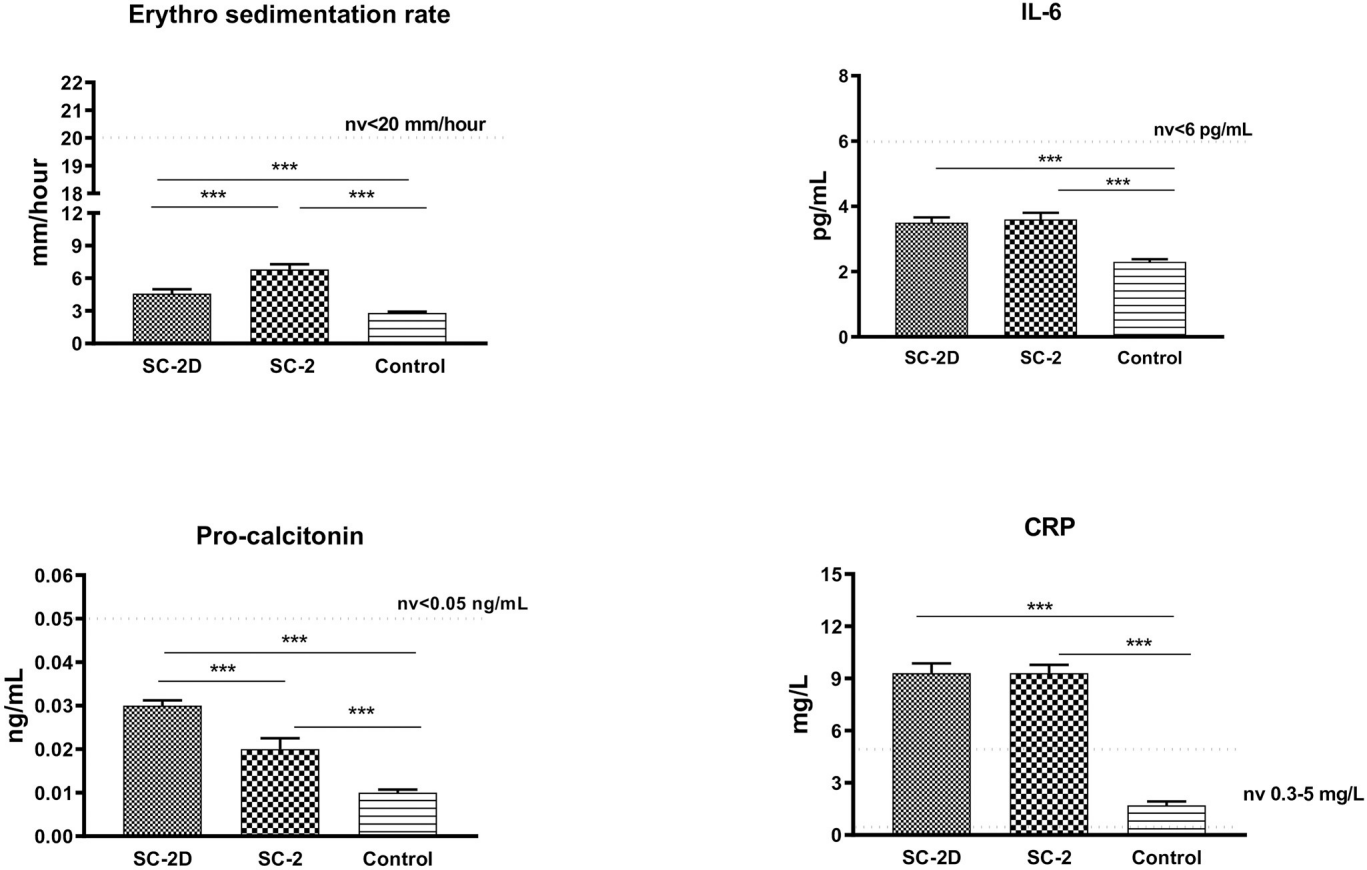
Inflammation biomarkers serum levels. Clinical biochemistry data on interleukin-6 (IL-6), C-reactive protein (CRP), erythrocyte sedimentation rate (ESR), and procalcitonin in Group 1: SC-2D, Group 2: SC-2, and Group 3: Control. Results are shown as mean ± SD. The analysis of variance test was performed to evaluate the differences between groups. ***P<0.01. P values remained significant after Bonferroni comparisons (P<0.01).

**Fig 4:**
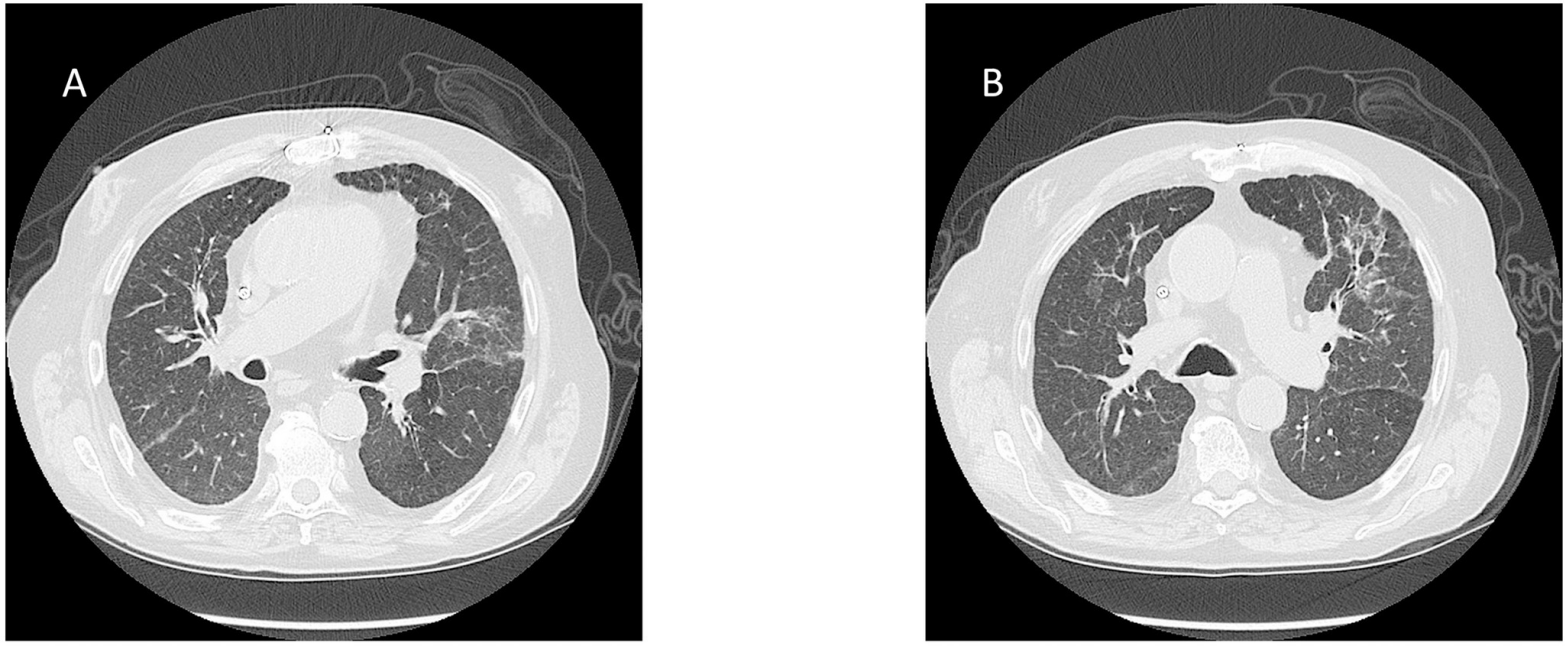
Computed tomography (CT) of two patients infected with SARS-CoV-2 with (Group 1, SC-2D) (A) and without (Group 2, SC-2) (B) dyspnea. In both images, interlobular septal thickening and patchy ground-glass opacities are evident.

Inflammatory biomarkers (IL-6, CRP, ESR, and procalcitonin) were evaluated in all groups. An increase in CRP levels outside the range was found in Group 1 and Group 2, but not in Group 3 (Fig 3). Finally, radiological examination confirmed the involvement of the chest in SARS-CoV-2 infected patients (Fig 4).

## 4. Discussion

Currently, the world continues to face the COVID-19 pandemic. To date, the evaluation of soluble factors has supported the early diagnosis or management of COVID-19 patients [11–13]. Although SARS-CoV-2 infection is known to greatly affect the lung tissue, NSE levels during infection have never been reported. Moreover, the multifunctional role of NSE in lung disease is well established. Zhong et al. [14] described increased NSE levels in animal models of acute lung injury following exposure to lipopolysaccharide. Sheppard et al. [15] reported that asbestos could induce tissue expression of NSE in lung endocrine cells, while Fang et al. [16] observed an NSE increase in 11 patients with pulmonary alveolar proteinosis, a rare interstitial lung disease with a decrease in lung ventilation function. Finally, in patients with tuberculosis, NSE levels were elevated in 91.66% of cases, suggesting the highest sensitivity that could be useful for the diagnosis of smear-negative tuberculosis and acute military tuberculosis secondary to tuberculous meningitis [8, 17]. Recently, a four-fold increase in NSE was reported in the CSF of a patient with SARS-CoV-2 infection [18]. Based on this single observation, the authors suggested that NSE could be a diagnostic/prognostic biomarker for neuroinflammation in patients infected with SARS-CoV-2, and particularly in patients with neurological symptoms. Here we explored the novel aspect of NSE in patients who developed dyspnea during COVID-19 with lung failure. Moreover, NSE is known to promote the synthesis of proinflammatory mediators [19]. In this study, CRP significantly increased in both Groups 1 and 2, thus boosting the inflammatory environment. In previous studies of COVID-19, IL-6 was reported to be an independent and significant predictor of disease severity and death [20], and Sabaka et al. [21] reported that plasma IL-6 values >24 pg/mL predicted the development of hypoxemia with a high sensitivity and specificity of 100% and 88.9%, respectively. In contrast, we did not find an increase in IL-6 in SARS-CoV-2 infected patients, even in critically ill patients, in this study. This could be related to the small sample size and lower viral load. Moreover, IL-6 levels in COVID-19 patients are largely variable, since multiple measurements of serum IL-6 levels have shown fluctuations both within the course of infection and within the same patient [22]. Therefore, it is possible that in a large sample of patients with a high viral load, higher levels of IL-6 (>5 pg/mL) could be identified. Furthermore, we did not find an increase in procalcitonin, which is related to the absence of systemic sepsis in the enrolled patients. Therefore, we cannot exclude an increase in this biomarker in SARS-CoV-2 infected patients with severe bacterial infections and sepsis.

In conclusion, this is the first study to show high serum NSE levels in patients with SARS-CoV-2 infection, and particularly in those with dyspnea. We believe that the introduction of NSE in a panel of soluble biomarkers could constitute a powerful additional strategy for disease monitoring in COVID-19 patients, even though biomarker changes should be constantly monitored. Further studies should be performed to validate our observations in a large cohort. However, these data allow for further investigation of the potential role of NSE in respiratory infectious diseases.
